# Supplementary material for: RNA structure prediction using positive and negative evolutionary information
Source: PLoS Comput Biol. 2020 Oct 30;16(10):e1008387. doi: 10.1371/journal.pcbi.1008387 (PMC7657543; doi:10.1371/journal.pcbi.1008387)
Supplement: S1 File — Includes alignments, and source code. (GZ) [file pcbi.1008387.s008.tar.gz › supplemental_material/results/Figures/RF00005_tRNA.fold.R2R.sto.pdf]

RF00005_tRNA

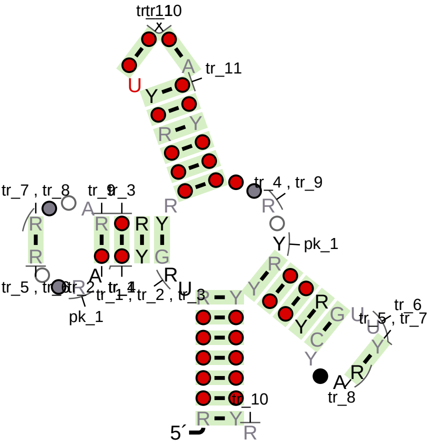

pk_1

R – Y

tr_1

R – ●

tr_10

● – R

tr_11

● – ●

tr_2

R – ●

tr_3

R – ●

tr_4

● – R

tr_5

R – Y

tr_6

R – U

tr_7

R – Y

tr_8

R – R

tr_9

R – R
